# 4-(4-Chloro­phen­yl)-6-meth­oxy-2,2′-bipyridine-5-carbonitrile

**DOI:** 10.1107/S1600536809012409

**Published:** 2009-04-08

**Authors:** P. Ramesh, S. S. Sundaresan, P. Thirumurugan, Paramasivan T. Perumal, M. N. Ponnuswamy

**Affiliations:** a Department of Physics, Presidency College (Autonomous), Chennai 600 005, India; b Centre of Advanced Study in Crystallography and Biophysics, University of Madras, Guindy Campus, Chennai 600 025, India; c Organic Chemistry Division, Central Leather Research Institute, Adyar, Chennai 600 020, India

## Abstract

There are two independent mol­ecules in the asymmetric unit of the title compound, C_18_H_12_ClN_3_O. The two pyridine rings are almost coplanar [dihedral angles between the rings: 2.87 (15) and 5.36 (16)°] while the chloro­phenyl rings are twisted out of the plane of the adjacent bipyridine ring by 44.1 (1) and 43.8 (1)° in the two mol­ecules. The crystal packing is stabilized by C—H⋯N and C—H⋯Cl inter­actions.

## Related literature

Pyridine derivatives possess phospho­diesterase-inhibiting (Heintzelman *et al.*, 2003*a* ▶,*b* ▶), anti­fungal (Cook *et al.*, 2004*a* ▶,*b* ▶), anti­fertility (Upton *et al.*, 2000 ▶) and anti­arrhythmic activities (Ellefson *et al.*, 1978 ▶). For hydrogen-bond motifs, see: Bernstein *et al.* (1995 ▶).
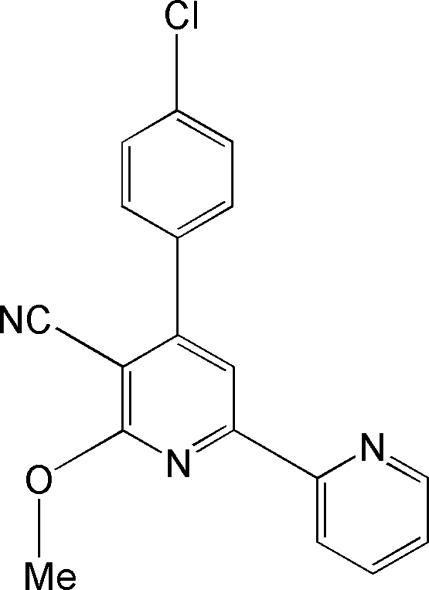


## Experimental

### 

#### Crystal data

C_18_H_12_ClN_3_O
*M* *_r_* = 321.76
Monoclinic, 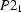

*a* = 9.5869 (5) Å
*b* = 13.8761 (7) Å
*c* = 12.2124 (6) Å
β = 106.896 (2)°
*V* = 1554.47 (14) Å^3^
*Z* = 4
Mo *K*α radiation
μ = 0.25 mm^−1^
*T* = 293 K
0.30 × 0.25 × 0.20 mm

#### Data collection

Bruker Kappa APEXII area-detector diffractometer
Absorption correction: multi-scan (*SADABS*; Sheldrick, 2001 ▶) *T*_min_ = 0.927, *T*_max_ = 0.951
17581 measured reflections
6950 independent reflections
4300 reflections with *I* > 2σ(*I*)
*R*_int_ = 0.035

#### Refinement

*R*[*F* ^2^ > 2σ(*F* ^2^)] = 0.043
*wR*(*F* ^2^) = 0.113
*S* = 1.00
6950 reflections
417 parameters
1 restraint
H-atom parameters constrained
Δρ_max_ = 0.24 e Å^−3^
Δρ_min_ = −0.18 e Å^−3^
Absolute structure: Flack (1983 ▶), 3268 Friedel pairs
Flack parameter: 0.05 (6)

### 

Data collection: *APEX2* (Bruker, 2004 ▶); cell refinement: *SAINT* (Bruker, 2004 ▶); data reduction: *SAINT*; program(s) used to solve structure: *SIR92* (Altomare *et al.*, 1993 ▶); program(s) used to refine structure: *SHELXL97* (Sheldrick, 2008 ▶); molecular graphics: *ORTEP-3* (Farrugia, 1997 ▶); software used to prepare material for publication: *SHELXL97* and *PLATON* (Spek, 2009 ▶).

## Supplementary Material

Crystal structure: contains datablocks global, I. DOI: 10.1107/S1600536809012409/bt2899sup1.cif

Structure factors: contains datablocks I. DOI: 10.1107/S1600536809012409/bt2899Isup2.hkl

Additional supplementary materials:  crystallographic information; 3D view; checkCIF report

## Figures and Tables

**Table 1:** Hydrogen-bond geometry (Å, °)

*D*—H⋯*A*	*D*—H	H⋯*A*	*D*⋯*A*	*D*—H⋯*A*
C11—H11⋯N9′^i^	0.93	2.58	3.321 (4)	137
C21—H21⋯Cl1^ii^	0.93	2.80	3.597 (3)	144
C11′—H11′⋯N9^iii^	0.93	2.60	3.349 (4)	138
C21′—H21′⋯Cl1′^ii^	0.93	2.71	3.475 (3)	140

Symmetry codes: (i) 
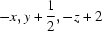
; (ii) 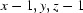; (iii) 
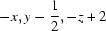
.

**Fig. 1.**
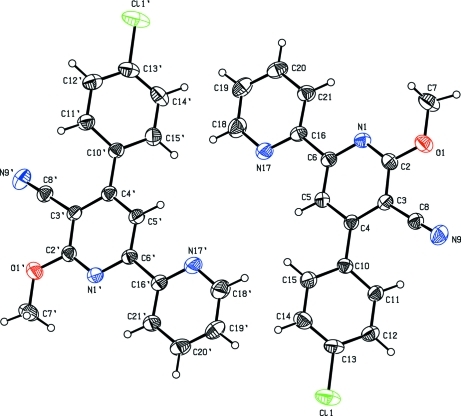
Perspective view of the molecule showing the displacement ellipsoids at 50% probability level. The H atoms are shown as small circles of arbitrary radii.

**Fig. 2.**
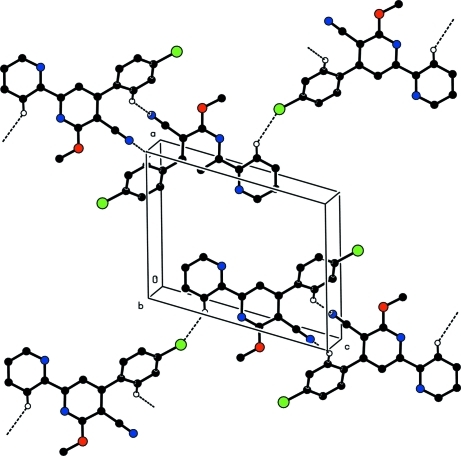
The crystal packing of the molecules viewed down b axis. H atoms not involved in hydrogen bonding have been omitted for clarity.

## supplementary crystallographic information

### Comment

Pyridine derivatives possess phosphodiesterase inhibiting (Heintzelman *et al.*, 2003*a*,*b*), antifungal (Cook *et al.*, 2004*a*,*b*), antifertility (Upton *et al.*,2000)  and antiarrhythmic activities (Ellefson *et al.*, 1978). The crystallographic study was useful to ascertain the molecular conformation.

The *ORTEP* plot of the molecule is shown in Fig.1. There are two crystallographically independent molecules in the asymmetric unit. The two pyridine rings lie in the same plane which is evidenced from the dihedral angles of 2.87 (15)° and 5.36 (16)° for the molecules 1 & 2 respectively. The planar chlorophenyl rings are twisted away from the bipyridine ring by 44.1 (1)° for (molecule 1) and 43.8 (1)° for (molecule 2), respectively. The bond angles of C3—C8—N9 (177.9 (4))° and C3'-C8'-N9' (178.3 (3))° show linearity of the cyano group, a feature observed in carbonitrile compounds.

The crystal packing is controlled by C—H···N and C—H···Cl intermolecular interactions in addition to van der Waals forces. Atoms C11 and C11' at (*x*, *y*, *z*) donate one proton each to N9' (-*x*,+*y* + 1/2,-*z* + 2) and N9 (-*x*,+*y* - 1/2,-*z* + 2) which connect the molecules to form a dimer with a graph-set-motiff *R*^2^_2_(14) (Bernstein *et al.*, 1995). These dimers are linked through intermolecular C21—H21···Cl1 hydrogen bond chain running along *c* axis which is shown in Fig. 2.

### Experimental

A mixture of *p*-chlorobenzaldehyde (1 eq), 2-acetyl pyridine and sodium hydroxide (1.2 eq) in methanol was refluxed for 30 min. After that malanonitrile (1 eq) was added and the reaction was continued to 3 h. With the completion of the reaction (as monitored by TLC), it was poured into water and extracted with ethyl acetate. The organic layer was dried over sodium sulfate and concentrated under vacuo. The crude product was chromatographed and isolated in 76% yield (90:10, petroleum ether: ethyl acetate). The compound was recrystallized in ethanol.

### Refinement

All H atoms were positioned geometrically (C—H=0.93–0.97 Å) and allowed to ride on their parent atoms, with *U*_iso_(H) = 1.5*U*_eq_(C) for methyl H, 1.2*U*_eq_(C) for other H atoms.

### Crystal data

**Table:**

C_18_H_12_ClN_3_O	*F*(000) = 664
*M**_r_* = 321.76	*D*_x_ = 1.375 Mg m^−^^3^
Monoclinic, *P*2_1_	Mo *K*α radiation, λ = 0.71073 Å
Hall symbol: P 2yb	Cell parameters from 3721 reflections
*a* = 9.5869 (5) Å	θ = 1.7–27.5°
*b* = 13.8761 (7) Å	µ = 0.25 mm^−^^1^
*c* = 12.2124 (6) Å	*T* = 293 K
β = 106.896 (2)°	Block, colorless
*V* = 1554.47 (14) Å^3^	0.30 × 0.25 × 0.20 mm
*Z* = 4	

### Data collection

**Table:**

Bruker Kappa APEXII area-detector diffractometer	6950 independent reflections
Radiation source: fine-focus sealed tube	4300 reflections with *I* > 2σ(*I*)
graphite	*R*_int_ = 0.035
ω and φ scans	θ_max_ = 27.5°, θ_min_ = 1.7°
Absorption correction: multi-scan (*SADABS*; Sheldrick, 2001)	*h* = −12→12
*T*_min_ = 0.927, *T*_max_ = 0.951	*k* = −17→17
17581 measured reflections	*l* = −15→15

### Refinement

**Table:**

Refinement on *F*^2^	Secondary atom site location: difference Fourier map
Least-squares matrix: full	Hydrogen site location: inferred from neighbouring sites
*R*[*F*^2^ > 2σ(*F*^2^)] = 0.043	H-atom parameters constrained
*wR*(*F*^2^) = 0.113	*w* = 1/[σ^2^(*F*_o_^2^) + (0.0461*P*)^2^ + 0.1394*P*] where *P* = (*F*_o_^2^ + 2*F*_c_^2^)/3
*S* = 1.00	(Δ/σ)_max_ = 0.002
6950 reflections	Δρ_max_ = 0.24 e Å^−^^3^
417 parameters	Δρ_min_ = −0.18 e Å^−^^3^
1 restraint	Absolute structure: Flack (1983), 3268 Friedel pairs
Primary atom site location: structure-invariant direct methods	Flack parameter: 0.05 (6)

### Special details

**Table:**

Geometry. All e.s.d.'s (except the e.s.d. in the dihedral angle between two l.s. planes) are estimated using the full covariance matrix. The cell e.s.d.'s are taken into account individually in the estimation of e.s.d.'s in distances, angles and torsion angles; correlations between e.s.d.'s in cell parameters are only used when they are defined by crystal symmetry. An approximate (isotropic) treatment of cell e.s.d.'s is used for estimating e.s.d.'s involving l.s. planes.
Refinement. Refinement of *F*^2^ against ALL reflections. The weighted *R*-factor *wR* and goodness of fit *S* are based on *F*^2^, conventional *R*-factors *R* are based on *F*, with *F* set to zero for negative *F*^2^. The threshold expression of *F*^2^ > σ(*F*^2^) is used only for calculating *R*-factors(gt) *etc*. and is not relevant to the choice of reflections for refinement. *R*-factors based on *F*^2^ are statistically about twice as large as those based on *F*, and *R*- factors based on ALL data will be even larger.

### Fractional atomic coordinates and isotropic or equivalent isotropic displacement parameters (Å^2^)

**Table:**

	*x*	*y*	*z*	*U*_iso_*/*U*_eq_	
C2'	−0.0524 (3)	0.2747 (2)	0.5616 (2)	0.0453 (7)	
C2	−0.1641 (3)	0.5282 (2)	0.7627 (2)	0.0485 (7)	
C3'	0.0529 (3)	0.2755 (2)	0.66886 (19)	0.0438 (6)	
C3	−0.0610 (3)	0.5309 (2)	0.86978 (19)	0.0448 (6)	
C4'	0.1994 (3)	0.2704 (2)	0.67256 (19)	0.0450 (6)	
C4	0.0852 (3)	0.53628 (19)	0.87589 (19)	0.0427 (6)	
C5	0.1208 (3)	0.5349 (2)	0.77260 (19)	0.0452 (6)	
H5	0.2177	0.5373	0.7726	0.054*	
C5'	0.2306 (3)	0.2696 (2)	0.56900 (19)	0.0467 (6)	
H5'	0.3271	0.2672	0.5679	0.056*	
C6	0.0109 (3)	0.5301 (2)	0.6710 (2)	0.0439 (6)	
C6'	0.1199 (3)	0.2723 (2)	0.4671 (2)	0.0430 (6)	
C7'	−0.3014 (3)	0.2777 (3)	0.4542 (2)	0.0730 (9)	
H7D	−0.2843	0.3307	0.4092	0.110*	
H7F	−0.3957	0.2845	0.4658	0.110*	
H7E	−0.2977	0.2183	0.4147	0.110*	
C7	−0.4109 (3)	0.5274 (3)	0.6510 (2)	0.0769 (10)	
H7A	−0.3862	0.5766	0.6046	0.115*	
H7B	−0.5054	0.5407	0.6598	0.115*	
H7C	−0.4125	0.4659	0.6147	0.115*	
C8'	0.0057 (3)	0.2856 (2)	0.7694 (2)	0.0506 (7)	
C8	−0.1112 (3)	0.5223 (2)	0.9698 (2)	0.0509 (7)	
C10	0.2010 (3)	0.5420 (2)	0.9863 (2)	0.0452 (7)	
C10'	0.3164 (3)	0.2671 (2)	0.78201 (19)	0.0451 (6)	
C11	0.1893 (3)	0.6056 (2)	1.0710 (2)	0.0488 (7)	
H11	0.1081	0.6455	1.0575	0.059*	
C11'	0.3071 (3)	0.2046 (2)	0.8686 (2)	0.0540 (7)	
H11'	0.2262	0.1646	0.8573	0.065*	
C12'	0.4158 (3)	0.2011 (2)	0.9709 (2)	0.0601 (8)	
H12'	0.4089	0.1587	1.0281	0.072*	
C12	0.2950 (3)	0.6106 (2)	1.1738 (2)	0.0540 (7)	
H12	0.2867	0.6538	1.2298	0.065*	
C13'	0.5332 (3)	0.2601 (3)	0.9877 (2)	0.0609 (9)	
C13	0.4138 (3)	0.5507 (3)	1.1932 (2)	0.0565 (8)	
C14	0.4309 (3)	0.4900 (3)	1.1102 (2)	0.0646 (9)	
H14	0.5141	0.4520	1.1233	0.078*	
C14'	0.5486 (3)	0.3210 (3)	0.9047 (3)	0.0661 (9)	
H14'	0.6307	0.3600	0.9172	0.079*	
C15'	0.4401 (3)	0.3240 (2)	0.8012 (2)	0.0567 (8)	
H15'	0.4504	0.3648	0.7436	0.068*	
C15	0.3241 (3)	0.4854 (2)	1.0067 (2)	0.0572 (8)	
H15	0.3351	0.4438	0.9501	0.069*	
C16	0.0424 (3)	0.5259 (2)	0.5593 (2)	0.0454 (7)	
C16'	0.1494 (3)	0.2756 (2)	0.3547 (2)	0.0464 (6)	
C18'	0.3136 (4)	0.2815 (3)	0.2542 (3)	0.0821 (11)	
H18'	0.4099	0.2807	0.2524	0.098*	
C18	0.2082 (4)	0.5207 (3)	0.4595 (3)	0.0717 (10)	
H18	0.3052	0.5202	0.4594	0.086*	
C19	0.1032 (4)	0.5161 (2)	0.3559 (3)	0.0662 (10)	
H19	0.1284	0.5128	0.2880	0.079*	
C19'	0.2062 (4)	0.2900 (3)	0.1528 (2)	0.0715 (10)	
H19'	0.2299	0.2950	0.0844	0.086*	
C20'	0.0658 (4)	0.2910 (3)	0.1527 (2)	0.0643 (9)	
H20'	−0.0091	0.2968	0.0848	0.077*	
C20	−0.0384 (4)	0.5166 (3)	0.3552 (2)	0.0629 (9)	
H20	−0.1127	0.5133	0.2866	0.075*	
C21	−0.0705 (3)	0.5222 (2)	0.4585 (2)	0.0554 (7)	
H21	−0.1668	0.5236	0.4601	0.067*	
C21'	0.0364 (3)	0.2832 (2)	0.2554 (2)	0.0550 (8)	
H21'	−0.0596	0.2831	0.2581	0.066*	
N1	−0.1311 (2)	0.52769 (17)	0.66537 (16)	0.0476 (6)	
N1'	−0.0218 (2)	0.27322 (17)	0.46358 (16)	0.0477 (5)	
N9'	−0.0333 (3)	0.2959 (2)	0.8486 (2)	0.0696 (8)	
N9	−0.1527 (3)	0.5130 (2)	1.0466 (2)	0.0711 (8)	
N17'	0.2879 (3)	0.2742 (2)	0.35573 (18)	0.0655 (7)	
N17	0.1810 (3)	0.5257 (2)	0.56052 (18)	0.0594 (7)	
O1	−0.3049 (2)	0.52612 (18)	0.76094 (15)	0.0623 (6)	
O1'	−0.1919 (2)	0.27689 (18)	0.56248 (16)	0.0617 (6)	
Cl1'	0.66572 (10)	0.25841 (9)	1.11925 (7)	0.0970 (4)	
Cl1	0.54493 (9)	0.55353 (8)	1.32596 (6)	0.0888 (3)	

### Atomic displacement parameters (Å^2^)

**Table:**

	*U*^11^	*U*^22^	*U*^33^	*U*^12^	*U*^13^	*U*^23^
C2'	0.0455 (16)	0.0402 (16)	0.0507 (15)	−0.0023 (15)	0.0149 (12)	0.0032 (13)
C2	0.0441 (15)	0.0460 (17)	0.0545 (15)	−0.0014 (14)	0.0130 (12)	0.0042 (14)
C3'	0.0524 (16)	0.0416 (16)	0.0372 (13)	−0.0055 (15)	0.0126 (11)	0.0029 (14)
C3	0.0522 (16)	0.0423 (15)	0.0408 (13)	0.0036 (14)	0.0149 (11)	0.0003 (13)
C4'	0.0522 (15)	0.0395 (15)	0.0422 (13)	−0.0043 (15)	0.0121 (11)	0.0023 (14)
C4	0.0472 (15)	0.0382 (15)	0.0422 (13)	0.0042 (13)	0.0120 (11)	−0.0021 (13)
C5	0.0436 (14)	0.0473 (16)	0.0448 (13)	0.0024 (13)	0.0130 (10)	−0.0011 (13)
C5'	0.0505 (15)	0.0454 (16)	0.0433 (13)	0.0005 (14)	0.0122 (11)	0.0043 (13)
C6	0.0504 (16)	0.0392 (15)	0.0416 (13)	−0.0009 (13)	0.0128 (11)	−0.0008 (13)
C6'	0.0496 (16)	0.0355 (14)	0.0427 (13)	0.0009 (14)	0.0113 (11)	0.0023 (13)
C7'	0.0506 (17)	0.090 (3)	0.0700 (19)	0.002 (2)	0.0034 (14)	0.000 (2)
C7	0.0470 (17)	0.102 (3)	0.074 (2)	−0.010 (2)	0.0058 (14)	0.011 (2)
C8'	0.0565 (18)	0.0444 (19)	0.0512 (16)	−0.0023 (16)	0.0161 (14)	0.0052 (14)
C8	0.0524 (16)	0.0514 (18)	0.0482 (15)	0.0058 (15)	0.0137 (12)	−0.0002 (15)
C10	0.0432 (15)	0.0486 (17)	0.0427 (13)	−0.0014 (14)	0.0108 (11)	0.0027 (14)
C10'	0.0512 (15)	0.0468 (15)	0.0377 (12)	−0.0019 (14)	0.0135 (10)	−0.0030 (13)
C11	0.0535 (17)	0.0529 (17)	0.0414 (14)	0.0028 (14)	0.0159 (12)	−0.0007 (13)
C11'	0.0587 (18)	0.0557 (18)	0.0451 (15)	−0.0016 (14)	0.0110 (12)	0.0012 (14)
C12'	0.068 (2)	0.066 (2)	0.0444 (15)	0.0144 (17)	0.0133 (14)	0.0025 (15)
C12	0.0605 (18)	0.0609 (19)	0.0416 (14)	−0.0107 (16)	0.0167 (13)	−0.0050 (13)
C13'	0.0528 (18)	0.076 (2)	0.0464 (15)	0.0167 (18)	0.0034 (13)	−0.0115 (17)
C13	0.0516 (17)	0.069 (2)	0.0411 (14)	−0.0138 (17)	0.0015 (12)	0.0058 (16)
C14	0.0552 (19)	0.068 (2)	0.0638 (19)	0.0076 (17)	0.0063 (16)	0.0046 (18)
C14'	0.0426 (17)	0.078 (2)	0.074 (2)	−0.0076 (16)	0.0120 (16)	−0.0202 (19)
C15'	0.0550 (18)	0.060 (2)	0.0569 (17)	−0.0061 (15)	0.0200 (14)	−0.0035 (15)
C15	0.0569 (18)	0.057 (2)	0.0541 (16)	0.0098 (15)	0.0107 (14)	−0.0035 (15)
C16	0.0549 (17)	0.0386 (16)	0.0418 (14)	−0.0049 (15)	0.0124 (12)	−0.0012 (13)
C16'	0.0569 (16)	0.0394 (15)	0.0427 (13)	−0.0013 (15)	0.0143 (11)	−0.0003 (13)
C18'	0.076 (2)	0.122 (3)	0.0567 (18)	0.010 (2)	0.0321 (16)	0.009 (2)
C18	0.077 (2)	0.083 (3)	0.065 (2)	−0.008 (2)	0.0367 (18)	−0.004 (2)
C19	0.097 (3)	0.059 (2)	0.0525 (18)	−0.012 (2)	0.0378 (18)	−0.0001 (16)
C19'	0.094 (3)	0.081 (3)	0.0451 (16)	0.003 (2)	0.0294 (17)	0.0030 (18)
C20'	0.083 (2)	0.065 (2)	0.0393 (15)	−0.0019 (19)	0.0100 (15)	−0.0059 (16)
C20	0.085 (2)	0.058 (2)	0.0422 (16)	−0.0044 (19)	0.0135 (16)	0.0041 (15)
C21	0.0601 (18)	0.0571 (19)	0.0465 (15)	0.0019 (16)	0.0113 (12)	0.0031 (14)
C21'	0.0580 (17)	0.062 (2)	0.0438 (15)	−0.0013 (16)	0.0121 (12)	−0.0027 (15)
N1	0.0496 (13)	0.0515 (15)	0.0400 (11)	−0.0026 (12)	0.0102 (9)	0.0012 (11)
N1'	0.0509 (13)	0.0470 (13)	0.0436 (12)	−0.0002 (13)	0.0113 (10)	0.0018 (11)
N9'	0.093 (2)	0.0659 (19)	0.0600 (16)	−0.0049 (15)	0.0382 (15)	0.0010 (14)
N9	0.0778 (18)	0.084 (2)	0.0598 (15)	0.0023 (16)	0.0336 (13)	−0.0006 (15)
N17'	0.0601 (16)	0.089 (2)	0.0499 (13)	0.0088 (17)	0.0201 (11)	0.0099 (15)
N17	0.0592 (15)	0.0710 (18)	0.0500 (13)	−0.0052 (15)	0.0190 (11)	−0.0064 (14)
O1	0.0460 (11)	0.0844 (17)	0.0555 (11)	−0.0024 (12)	0.0132 (9)	0.0104 (12)
O1'	0.0462 (11)	0.0785 (16)	0.0605 (12)	0.0000 (12)	0.0160 (9)	−0.0005 (12)
Cl1'	0.0709 (5)	0.1312 (9)	0.0664 (5)	0.0322 (6)	−0.0157 (4)	−0.0216 (6)
Cl1	0.0726 (5)	0.1207 (8)	0.0551 (4)	−0.0156 (5)	−0.0101 (3)	0.0127 (5)

### Geometric parameters (Å, °)

**Table:**

C2'—N1'	1.313 (3)	C11—H11	0.9300
C2'—O1'	1.341 (3)	C11'—C12'	1.375 (3)
C2'—C3'	1.403 (3)	C11'—H11'	0.9300
C2—N1	1.316 (3)	C12'—C13'	1.358 (5)
C2—O1	1.344 (3)	C12'—H12'	0.9300
C2—C3	1.391 (3)	C12—C13	1.374 (4)
C3'—C4'	1.394 (3)	C12—H12	0.9300
C3'—C8'	1.433 (4)	C13'—C14'	1.361 (5)
C3—C4	1.384 (3)	C13'—Cl1'	1.735 (3)
C3—C8	1.442 (4)	C13—C14	1.364 (4)
C4'—C5'	1.382 (3)	C13—Cl1	1.739 (3)
C4'—C10'	1.475 (3)	C14—C15	1.378 (4)
C4—C5	1.399 (3)	C14—H14	0.9300
C4—C10	1.478 (3)	C14'—C15'	1.384 (4)
C5—C6	1.376 (3)	C14'—H14'	0.9300
C5—H5	0.9300	C15'—H15'	0.9300
C5'—C6'	1.382 (3)	C15—H15	0.9300
C5'—H5'	0.9300	C16—N17	1.324 (3)
C6—N1	1.343 (3)	C16—C21	1.384 (3)
C6—C16	1.481 (3)	C16'—N17'	1.325 (3)
C6'—N1'	1.346 (3)	C16'—C21'	1.376 (3)
C6'—C16'	1.481 (3)	C18'—N17'	1.337 (4)
C7'—O1'	1.430 (3)	C18'—C19'	1.366 (4)
C7'—H7D	0.9600	C18'—H18'	0.9300
C7'—H7F	0.9600	C18—N17	1.335 (3)
C7'—H7E	0.9600	C18—C19	1.370 (4)
C7—O1	1.430 (3)	C18—H18	0.9300
C7—H7A	0.9600	C19—C20	1.355 (4)
C7—H7B	0.9600	C19—H19	0.9300
C7—H7C	0.9600	C19'—C20'	1.346 (4)
C8'—N9'	1.143 (4)	C19'—H19'	0.9300
C8—N9	1.127 (3)	C20'—C21'	1.367 (4)
C10—C15	1.379 (4)	C20'—H20'	0.9300
C10—C11	1.388 (4)	C20—C21	1.384 (4)
C10'—C15'	1.387 (4)	C20—H20	0.9300
C10'—C11'	1.391 (4)	C21—H21	0.9300
C11—C12	1.368 (4)	C21'—H21'	0.9300
			
N1'—C2'—O1'	119.7 (2)	C11'—C12'—H12'	120.3
N1'—C2'—C3'	124.1 (2)	C11—C12—C13	118.9 (3)
O1'—C2'—C3'	116.2 (2)	C11—C12—H12	120.5
N1—C2—O1	119.3 (2)	C13—C12—H12	120.5
N1—C2—C3	123.9 (2)	C12'—C13'—C14'	121.7 (3)
O1—C2—C3	116.8 (2)	C12'—C13'—Cl1'	118.9 (3)
C4'—C3'—C2'	118.4 (2)	C14'—C13'—Cl1'	119.3 (3)
C4'—C3'—C8'	122.9 (2)	C14—C13—C12	121.2 (2)
C2'—C3'—C8'	118.7 (2)	C14—C13—Cl1	119.8 (3)
C4—C3—C2	118.9 (2)	C12—C13—Cl1	119.0 (2)
C4—C3—C8	122.8 (2)	C13—C14—C15	119.5 (3)
C2—C3—C8	118.2 (2)	C13—C14—H14	120.2
C5'—C4'—C3'	117.0 (2)	C15—C14—H14	120.2
C5'—C4'—C10'	121.3 (2)	C13'—C14'—C15'	118.9 (3)
C3'—C4'—C10'	121.6 (2)	C13'—C14'—H14'	120.5
C3—C4—C5	117.3 (2)	C15'—C14'—H14'	120.5
C3—C4—C10	122.1 (2)	C14'—C15'—C10'	121.0 (3)
C5—C4—C10	120.5 (2)	C14'—C15'—H15'	119.5
C6—C5—C4	119.4 (2)	C10'—C15'—H15'	119.5
C6—C5—H5	120.3	C14—C15—C10	120.6 (3)
C4—C5—H5	120.3	C14—C15—H15	119.7
C4'—C5'—C6'	120.7 (2)	C10—C15—H15	119.7
C4'—C5'—H5'	119.7	N17—C16—C21	122.2 (2)
C6'—C5'—H5'	119.7	N17—C16—C6	117.5 (2)
N1—C6—C5	123.1 (2)	C21—C16—C6	120.3 (2)
N1—C6—C16	115.3 (2)	N17'—C16'—C21'	122.7 (2)
C5—C6—C16	121.6 (2)	N17'—C16'—C6'	116.9 (2)
N1'—C6'—C5'	122.2 (2)	C21'—C16'—C6'	120.4 (3)
N1'—C6'—C16'	115.6 (2)	N17'—C18'—C19'	123.6 (3)
C5'—C6'—C16'	122.1 (2)	N17'—C18'—H18'	118.2
O1'—C7'—H7D	109.5	C19'—C18'—H18'	118.2
O1'—C7'—H7F	109.5	N17—C18—C19	124.5 (3)
H7D—C7'—H7F	109.5	N17—C18—H18	117.7
O1'—C7'—H7E	109.5	C19—C18—H18	117.7
H7D—C7'—H7E	109.5	C20—C19—C18	118.1 (3)
H7F—C7'—H7E	109.5	C20—C19—H19	120.9
O1—C7—H7A	109.5	C18—C19—H19	120.9
O1—C7—H7B	109.5	C20'—C19'—C18'	119.5 (3)
H7A—C7—H7B	109.5	C20'—C19'—H19'	120.3
O1—C7—H7C	109.5	C18'—C19'—H19'	120.3
H7A—C7—H7C	109.5	C19'—C20'—C21'	118.1 (3)
H7B—C7—H7C	109.5	C19'—C20'—H20'	120.9
N9'—C8'—C3'	178.3 (4)	C21'—C20'—H20'	120.9
N9—C8—C3	177.9 (3)	C19—C20—C21	118.8 (3)
C15—C10—C11	118.5 (2)	C19—C20—H20	120.6
C15—C10—C4	120.7 (2)	C21—C20—H20	120.6
C11—C10—C4	120.8 (2)	C16—C21—C20	119.3 (3)
C15'—C10'—C11'	117.9 (2)	C16—C21—H21	120.4
C15'—C10'—C4'	121.6 (2)	C20—C21—H21	120.4
C11'—C10'—C4'	120.6 (2)	C20'—C21'—C16'	119.7 (3)
C12—C11—C10	121.2 (3)	C20'—C21'—H21'	120.2
C12—C11—H11	119.4	C16'—C21'—H21'	120.2
C10—C11—H11	119.4	C2—N1—C6	117.4 (2)
C12'—C11'—C10'	120.9 (3)	C2'—N1'—C6'	117.50 (19)
C12'—C11'—H11'	119.6	C16'—N17'—C18'	116.4 (3)
C10'—C11'—H11'	119.6	C16—N17—C18	117.1 (2)
C13'—C12'—C11'	119.5 (3)	C2—O1—C7	116.9 (2)
C13'—C12'—H12'	120.3	C2'—O1'—C7'	117.3 (2)
			
N1'—C2'—C3'—C4'	−2.8 (5)	C12—C13—C14—C15	−3.0 (5)
O1'—C2'—C3'—C4'	178.1 (3)	Cl1—C13—C14—C15	177.6 (2)
N1'—C2'—C3'—C8'	174.6 (3)	C12'—C13'—C14'—C15'	1.2 (5)
O1'—C2'—C3'—C8'	−4.5 (4)	Cl1'—C13'—C14'—C15'	−178.0 (2)
N1—C2—C3—C4	2.0 (4)	C13'—C14'—C15'—C10'	0.8 (5)
O1—C2—C3—C4	−178.0 (3)	C11'—C10'—C15'—C14'	−2.1 (4)
N1—C2—C3—C8	−174.4 (3)	C4'—C10'—C15'—C14'	179.4 (3)
O1—C2—C3—C8	5.6 (4)	C13—C14—C15—C10	0.5 (5)
C2'—C3'—C4'—C5'	3.1 (4)	C11—C10—C15—C14	1.8 (4)
C8'—C3'—C4'—C5'	−174.2 (3)	C4—C10—C15—C14	−179.2 (3)
C2'—C3'—C4'—C10'	−177.7 (3)	N1—C6—C16—N17	−178.4 (3)
C8'—C3'—C4'—C10'	5.1 (5)	C5—C6—C16—N17	0.8 (4)
C2—C3—C4—C5	−2.4 (4)	N1—C6—C16—C21	1.6 (4)
C8—C3—C4—C5	173.8 (3)	C5—C6—C16—C21	−179.2 (3)
C2—C3—C4—C10	178.3 (3)	N1'—C6'—C16'—N17'	−179.6 (3)
C8—C3—C4—C10	−5.4 (4)	C5'—C6'—C16'—N17'	1.2 (4)
C3—C4—C5—C6	1.1 (4)	N1'—C6'—C16'—C21'	2.5 (4)
C10—C4—C5—C6	−179.6 (3)	C5'—C6'—C16'—C21'	−176.7 (3)
C3'—C4'—C5'—C6'	−1.0 (4)	N17—C18—C19—C20	−0.1 (6)
C10'—C4'—C5'—C6'	179.7 (3)	N17'—C18'—C19'—C20'	−0.2 (7)
C4—C5—C6—N1	0.8 (4)	C18'—C19'—C20'—C21'	−0.1 (6)
C4—C5—C6—C16	−178.3 (3)	C18—C19—C20—C21	0.3 (5)
C4'—C5'—C6'—N1'	−1.8 (4)	N17—C16—C21—C20	1.1 (5)
C4'—C5'—C6'—C16'	177.4 (3)	C6—C16—C21—C20	−178.9 (3)
C4'—C3'—C8'—N9'	116 (12)	C19—C20—C21—C16	−0.8 (5)
C2'—C3'—C8'—N9'	−62 (12)	C19'—C20'—C21'—C16'	0.6 (5)
C4—C3—C8—N9	−124 (9)	N17'—C16'—C21'—C20'	−0.9 (5)
C2—C3—C8—N9	52 (9)	C6'—C16'—C21'—C20'	176.8 (3)
C3—C4—C10—C15	134.3 (3)	O1—C2—N1—C6	179.8 (3)
C5—C4—C10—C15	−45.0 (4)	C3—C2—N1—C6	−0.2 (4)
C3—C4—C10—C11	−46.8 (4)	C5—C6—N1—C2	−1.2 (4)
C5—C4—C10—C11	134.0 (3)	C16—C6—N1—C2	177.9 (2)
C5'—C4'—C10'—C15'	45.0 (4)	O1'—C2'—N1'—C6'	179.2 (3)
C3'—C4'—C10'—C15'	−134.2 (3)	C3'—C2'—N1'—C6'	0.1 (5)
C5'—C4'—C10'—C11'	−133.5 (3)	C5'—C6'—N1'—C2'	2.2 (4)
C3'—C4'—C10'—C11'	47.3 (4)	C16'—C6'—N1'—C2'	−177.0 (2)
C15—C10—C11—C12	−1.8 (4)	C21'—C16'—N17'—C18'	0.6 (5)
C4—C10—C11—C12	179.2 (3)	C6'—C16'—N17'—C18'	−177.2 (3)
C15'—C10'—C11'—C12'	1.4 (4)	C19'—C18'—N17'—C16'	0.0 (6)
C4'—C10'—C11'—C12'	179.9 (3)	C21—C16—N17—C18	−0.8 (5)
C10'—C11'—C12'—C13'	0.5 (4)	C6—C16—N17—C18	179.2 (3)
C10—C11—C12—C13	−0.6 (4)	C19—C18—N17—C16	0.3 (6)
C11'—C12'—C13'—C14'	−1.9 (5)	N1—C2—O1—C7	−2.6 (4)
C11'—C12'—C13'—Cl1'	177.3 (2)	C3—C2—O1—C7	177.5 (3)
C11—C12—C13—C14	3.0 (5)	N1'—C2'—O1'—C7'	−0.1 (4)
C11—C12—C13—Cl1	−177.5 (2)	C3'—C2'—O1'—C7'	179.1 (3)

### Hydrogen-bond geometry (Å, °)

**Table:**

*D*—H···*A*	*D*—H	H···*A*	*D*···*A*	*D*—H···*A*
C11—H11···N9'^i^	0.93	2.58	3.321 (4)	137
C21—H21···Cl1^ii^	0.93	2.80	3.597 (3)	144
C11'—H11'···N9^iii^	0.93	2.60	3.349 (4)	138
C21'—H21'···Cl1'^ii^	0.93	2.71	3.475 (3)	140

Symmetry codes: (i) −*x*, *y*+1/2, −*z*+2; (ii) *x*−1, *y*, *z*−1; (iii) −*x*, *y*−1/2, −*z*+2.
